# Reflection or Dependence: How AI Awareness Affects Employees’ In-Role and Extra-Role Performance?

**DOI:** 10.3390/bs15020128

**Published:** 2025-01-25

**Authors:** Heng Zhao, Long Ye, Ming Guo, Yanfang Deng

**Affiliations:** 1 School of Economics and Management, Beijing Jiaotong University, Beijing 100044, China; 2 School of Economics and Management, Lanzhou Jiaotong University, Lanzhou 730070, China

**Keywords:** AI awareness, perceived overqualification, reflection on AI usage, dependence on AI usage, in-role performance, extra-role performance, employee–AI collaboration

## Abstract

To address the challenges posed by AI technologies, an increasing number of organizations encourage or require employees to integrate AI into their work processes. Despite the extensive research that has explored AI applications in the workplace, limited attention has been paid to the role of AI awareness in shaping employees’ cognition, interaction behaviors with AI, and subsequent impacts. Drawing on self-construal theory, this study investigates how AI awareness influences employees’ in-role and extra-role performance. A multi-time-point analysis of data from 353 questionnaires reveals that employees’ AI awareness affects their perceived overqualification, which subsequently influences reflection on AI usage and dependence on AI usage, ultimately shaping their in-role and extra-role performance. Furthermore, employee–AI collaboration moderates the relationship between AI awareness and perceived overqualification. This study elucidates the mechanisms and boundary conditions through which AI awareness impacts employees’ performance, offering a more comprehensive perspective on AI awareness research and providing practical implications for promoting its positive effects while mitigating its negative consequences.

## 1. Introduction

With the rapid advances in AI technology involving data, algorithms, and computing power, employee AI awareness (AIA) has gradually become a focal point in examining the impact of artificial intelligence in the workplace (Parteka & Kordalska, 2023). AIA refers to employees’ perceptions and insights about how AI technologies could impact their future career prospects (Brougham & Haar, 2018; Kong et al., 2021). This concept has two sides: It encompasses not only workers’ positive assessments of the new opportunities that AI may offer society and even their own development but also their concerns about the inconveniences and potential risks of job replacement by AI technology. Therefore, employees’ AIA has both direct and indirect effects on their work performance, which is beginning to attract the attention of researchers (Chen et al., 2024; Spatola & Normand, 2021; Stein et al., 2020; Wang et al., 2022; Yin et al., 2024). Human–AI collaboration is becoming increasingly common, but research in the field of AI is still limited, mostly focusing on topics such as machine learning techniques and ethical considerations, aiming to design more powerful and convenient AI to assist humans (Vatankhah et al., 2024). Some scholars have called for a more comprehensive exploration of AI-related topics in future research to promote a more effective application of AI in various industries (Saydam et al., 2022; Wu & Zhang, 2024).

Perceived overqualification (POQ), as one of the most significant psychological states for employees (Erdogan & Bauer, 2009; Maynard et al., 2006), serves as a “bridge” for the causal relationships that this study seeks to uncover. In the United Kingdom, one-sixth of workers are considered to possess educational qualifications exceeding job requirements, with 58% of graduates employed in roles that do not require a university degree. Similarly, in the United States, one-fourth of employees holding a bachelor’s degree are regarded as overqualified for their jobs (David, 2017; Hultin et al., 2016). This employment environment, characterized by a large labor force and limited high-quality job opportunities, directly leads to the emergence of POQ among employees (Arvan et al., 2019; P. Liu et al., 2025; S. Liu et al., 2024). Furthermore, many companies prefer to hire highly qualified employees, as it is believed that overqualified individuals contribute greater initiative and superior performance to the organization (Luksyte & Spitzmueller, 2016). However, this often results in employees perceiving their abilities as underutilized, which negatively impacts their job satisfaction and performance (Ma et al., 2020).

As the impact of AI on the labor market sparks intense debate, employee interactions with AI are increasingly recognized as significant not only for individual career development and work experiences but also for job design, task analysis, and integration within organizational human resource management (Wu et al., 2024; Wu & Zhang, 2024). This study focuses on the following problems: What are the relationship and boundary conditions between AIA and POQ? How could organizations effectively address employees’ POQ? What are employees’ AI usage behaviors, and how do these behaviors influence in-role performance (IP) and extra-role performance (EP)?

To solve the above problems, this study makes several contributions. Firstly, this research provides a new perspective on AIA by using POQ to bridge the gap between AIA and performance, revealing how AIA indirectly affects EP and IP via POQ and the behavior of AI usage. Previous research has predominantly emphasized the negative effects of POQ, such as time theft behavior (Zhao & Ma, 2023), knowledge hiding (Khan et al., 2022; Li et al., 2022), and counterproductive work behaviors (Schreurs et al., 2020). However, a recent meta-analysis on POQ has uncovered an intriguing phenomenon: under specific management and human resource practices, employees with higher POQ often outperform their peers (Liao et al., 2024). Most existing studies on POQ are rooted in traditional work environments, with little attention paid to settings involving artificial intelligence applications. This study aims to address this gap by exploring the relationship between AIA and POQ.

Secondly, this study provides a tenable rationale for employees’ differing AI usage behaviors, grounded in self-construal theory. It reveals that independent self-construal drives reflection on AI usage (RAU), while relational self-construal fosters dependence on AI usage (DAU). In the workplace, AI is gradually evolving from a labor tool to a collaborative partner. The concepts of RAU and DAU proposed in this study align with the contextual trend of human-artificial intelligence collaboration. The focus of this research is not on the replacement or marginalization of employees by technology but rather on how employees can leverage AI to enhance both in-role and extra-role.

Thirdly, this study explored the moderating effect of employee–AI collaboration (EAC) between AIA and POQ. Previous research has identified external contextual factors and person–job misfit as key contributors to POQ. In the context of AI disruption, the extent of employee interaction with AI during the pursuit of work goals is likely a critical factor influencing the perception of overqualification (Erdogan & Bauer, 2009). Employees with high levels of collaboration with AI are more likely to reflect on the impact of AI disruption on themselves, including feelings of deprivation and job fit (Wu et al., 2024; Wu & Zhang, 2024). Conversely, employees with limited AI-related tasks may be less concerned about AI’s influence on their work. Thus, we propose that the relationship between AIA and POQ strengthens as the level of EAC increases.

**Figure 1:**
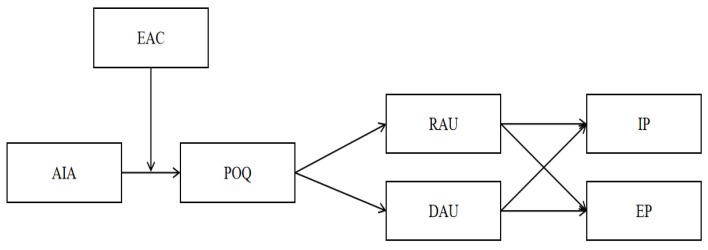
Theoretical model.

In conclusion, our study constructs a moderated chain-mediated model to deeply analyze the intrinsic mechanisms and boundary conditions of how AIA affects employees’ IP and EP. The findings are expected to provide practical guidance for organizational management, enabling organizations to better address the challenges posed by advancements in AI technology. The theoretical model of this study is shown in Figure 1.

## 2. Theoretical Framework and Hypotheses

### 2.1. Self-Construal Theory, Reflection on AI Usage, and Dependence on AI Usage

Self-construal theory, originally created by Markus and Kitayama in 1991, explains how individuals use their own perceptions to regulate their emotional and behavioral reactions. The theory makes a distinction between autonomous and interdependent perceptions of oneself (Markus & Kitayama, 1991). Individuals who have an independent self-construal see themselves as different from other people and place a high value on their own successes, goals, and independence. In this way of thinking, self-worth comes primarily from the expression of distinguishing characteristics and demonstrable abilities. Conversely, interdependent self-construal emphasizes the connection of people and social groups. In this form, personal identity is heavily influenced by group relationships and social harmony, with conduct frequently aimed at continuing favorable group dynamics. Individuals with interconnected self-concepts tend to obtain self-worth from teamwork and social support (Cross et al., 2011; Gudykunst et al., 1996; Singelis, 1994; Stapel & Koomen, 2001).

Self-construal has a profound impact on individuals’ behavioral, cognitive, and emotional responses (Hong & Chang, 2015; Poulin et al., 2021), and it similarly influences individuals’ behaviors regarding the use of AI. Employees with an independent self-construal who emphasize personal independence and self-expression tend to engage in self-reflection and decision-making rather than defining themselves based on the opinions of others (Kraus et al., 2012; Pöhlmann et al., 2007). Reflective behavior is characterized by active, sustained, and careful thoughts about problems, with self-reflection involving a deep analysis of past experiences, knowledge, and other informational resources, aimed at deliberately altering current and future behavior (Wood, 1996). Therefore, for individuals with an independent self-construal, RAU may be more prominent. These employees tend to critically evaluate AI’s output, focusing on the decision-making logic, limitations, and potential biases of the AI. They adjust their interactions with AI based on their own judgment to ensure that AI decisions align with both personal and organizational goals, demonstrating a high level of RAU. In contrast, employees with an interdependent self-construal, who emphasize coordination and cooperation within the group, are less likely to exhibit group bias, even when AI is viewed as an external entity. Instead, the similarity of AI tends to evoke a sense of closeness, and these employees demonstrate empathy toward AI (Chang et al., 2023; West et al., 2014). Coupled with AI’s advantages in data, algorithms, and computational power, these employees are more likely to exhibit DAU. These individuals tend to view AI as a critical tool for team collaboration and task completion and are more willing to accept AI’s suggestions and decisions to facilitate the achievement of group goals and improve work efficiency.

### 2.2. AI Awareness and Perceived Overqualification

Maynard et al. define POQ as a state of person–job misfit, where individuals perceive that the qualifications they possess, such as education, skills, and experience, exceed the demands of their current job (Maynard et al., 2006). Most research indicates that POQ is primarily related to person–job misfit and the negative psychological process of relative deprivation (Erdogan & Bauer, 2021). In the context of AI disruption, employees increasingly recognize the advantages of AI in task execution, including its efficiency, automation capabilities, and potential for solving complex problems. This awareness triggers a sense of insecurity about the possibility of AI replacing their current jobs in the future (He et al., 2023; Liang et al., 2022). Therefore, this study argues that AIA will have a significant impact on POQ.

First, from the perspective of person–job fit theory, AI assumes the demands of the job in terms of knowledge, skills, abilities, and other characteristics. AI’s learning and iteration speed far surpass that of an average employee, which may make employees feel it is difficult to achieve personal success, potentially leading to negative emotions such as anxiety (Wu et al., 2024). Inevitably, employees may perceive themselves as “overqualified” in terms of their knowledge and skills relative to the job, resulting in POQ. Moreover, from the perspective of relative deprivation theory, when individuals compare themselves to other reference points, they perceive themselves as being in a disadvantaged position, leading to feelings of deprivation and the experience of negative emotions such as anger and dissatisfaction. This is a subjective cognitive and emotional experience (Erdogan & Bauer, 2021; S. Liu et al., 2015). In the context of ongoing advancements in AI technology, companies are enhancing efficiency, reducing errors, and creating new job opportunities through automation and process optimization. The role of work is shifting from being a labor tool to becoming a collaborative partner. Employees with higher AIA inevitably compare and even compete with AI in the workplace. Since AI could perform many skilled human tasks effectively and efficiently (Makridakis, 2017; Nelson & Irwin, 2014), employees, when comparing themselves to AI, may perceive a disadvantaged position in the workplace. This comparison may lead to feelings of relative deprivation, where employees feel their skills and experience are no longer valued and their qualifications are underutilized, resulting in POQ.

**Hypothesis 1.**  *AIA will be positively related to POQ.*

### 2.3. Perceived Overqualification and Reflection on AI Usage and Dependence on AI Usage

Most research suggests that POQ leads to lower task performance and higher counterproductive behaviors (McKee-Ryan & Harvey, 2011; S. Liu et al., 2015). In contrast, emerging studies indicate that under certain conditions, POQ may have positive effects, such as increased career identification, improved performance, enhanced extra-role behaviors, and higher levels of knowledge sharing (Cheng et al., 2020; Deng et al., 2018; Luksyte et al., 2022; Zhang et al., 2016). Given that POQ could lead to both positive and negative outcomes, scholars have begun to investigate the contextual factors that explain these differing findings (Luksyte & Spitzmueller, 2016). In light of this, this study examines how employees’ interactions with Al technology are impacted by POQ spurred on by Al awareness amid Al disruption. The two separate behavioral patterns RAU and DAU that have been set out to describe the various reactions that employees show while interacting with AI technology are founded on self-construal theory.

From the perspective of independent self-construal, employees with an independent self-construal place greater emphasis on personal achievement and uniqueness. They tend to trust their own judgments and, when experiencing POQ, tend to have higher levels of self-efficacy, characterized by stronger confidence in their abilities and a greater desire to explore (Hu et al., 2015). Therefore, employees who feel overqualified are less likely to take Al’s conclusions at face value. Instead, they actively engage with Al’s working principles: ethical issues, privacy, and algorithmic biases in their work. They use their professional knowledge and experience to better understand the pros and cons of AI and reflect on its use.

On the contrary, employees with an interdependent self-concept place more emphasis on their relationships with others and their interactions with the social environment when defining their self. These individuals tend to be motivated to connect with others, value harmonious interactions with their environment, and have a strong sense of group identity (Esyun et al., 1985). When employees have an interdependent self-construal, they are very aware of the importance of collaboration and the use of external resources in a complex working environment. AI, with its efficient data processing, analysis, and decision support capabilities, serves as an effective external tool to help them complete tasks and achieve goals more effectively. Research has shown that employees with a high level of POQ often have a strong idea of their own capabilities (Erdogan & Bauer, 2021). As a result, employees are likely to feel that their qualifications are compatible and adaptable with the development of AI, giving them greater acceptance and control when engaging with AI. Therefore, employees are more likely to actively integrate AI into their work processes and rely more heavily on AI usage. Therefore, this research proposes the following hypothesis:

**Hypothesis 2a.**  *POQ will be positively related to RAU.*

**Hypothesis 2b.**  *POQ will be positively related to DAU.*

### 2.4. Reflection on AI Usage and In-Role and Extra-Role Performance

Work performance could be divided into IP and EP. IP, also referred to as task performance, refers to the work that employees complete within the scope of their job responsibilities as defined by the company, typically related to core technical tasks. Failure to complete this performance on time may result in direct losses to the company. EP, also known as contextual performance, refers to the additional benefits employees voluntarily bring to the organization, such as maintaining office harmony or optimizing team dynamics. These contributions are usually not included in the job description and are not solely measured by profit but rather reflect non-quantifiable organizational benefits. Out-of-role behaviors are also referred to as organizational citizenship behavior (Borman & Motowidlo, 1997; Kim et al., 2013).

Reflective thinking is particularly crucial for improving both IP and EP. Reflection helps employees better identify sources of information within the organization, enhances their ability to cope with environmental complexity, and ensures that decision-making is more complete and accurate, which in turn aids in recognizing and correcting potential errors, ultimately improving the overall quality of decisions (Ellis et al., 2006; Jong & Elfring, 2017; Schippers et al., 2013). According to Social Cognitive Theory (Bandura, 1991), most of the knowledge and skills required for task performance are acquired through observational learning rather than solely through trial and error based on direct experience. This learning process includes attention, memory retention, behavioral reproduction, and motivation for execution. Research has shown that employees with reflective thinking are more attentive to details, engage in deep thinking and mental simulations, and exhibit strong interest in how tasks are accomplished (Epstein et al., 1996), motivating them to actively learn new methods and skills. Reflection not only encourages employees to implement more efficient work plans and actions but could even generate useful ideas by breaking cognitive boundaries (Shin et al., 2019), thereby improving work performance (Dalal et al., 2010; Ellis & Davidi, 2005; Vance et al., 2007). This study suggests that as employees reflect on their use of AI, they become more immersed in their work, enhancing their work enthusiasm and out-of-role behaviors, which aligns AI more closely with their personal and organizational goals, thus increasing both IP and EP. Therefore, this research proposes the following hypotheses:

**Hypothesis 3a.**  *RAU will be positively related to IP.*

**Hypothesis 3b.**  *RAU will be positively related to EP.*

### 2.5. Dependence on AI Usage and In-Role and Extra-Role Performance

From the perspective of performance outcomes, IP serves as a critical representation of work output and measures employees’ ability to complete individual tasks (Borman & Motowidlo, 1997). Previous studies have shown that IP is influenced by factors such as interpersonal relationships and work roles (Ghosh et al., 2017). Regarding the impact of DAU on IP, such dependence reflects employees’ increased trust in AI, fostering harmonious working relationships between employees and AI systems (Glikson & Woolley, 2020; Kong et al., 2023). AI not only better learns human work processes, methods, and content but also more effectively understands employees’ behaviors and decisions, thereby facilitating the completion of in-role tasks. As AI systems are capable of processing large volumes of data and performing repetitive tasks, AI-assisted decision-support systems provide real-time analysis and recommendations. DAU signifies employees’ perception of resources, aligning with the conservation of resources theory, which posits that individuals are inclined to preserve valuable resources (Ghosh et al., 2017). To protect and retain these resources, employees tend to increase their work engagement, proactively take on responsibilities, and fully leverage the augmentative capabilities of AI, ultimately enhancing IP.

Regarding the impact of DAU on EP, such dependence allows employees to offload burdensome tasks, enabling them to focus on more complex and strategic work (Huang & Rust, 2018). Research has shown that AI, by reducing employee workload and improving the efficiency of work processes, could increase job satisfaction, which in turn encourages employees to proactively help colleagues and engage in organizational social activities (Huang & Rust, 2018). AI technologies could analyze and interpret data within work processes, providing employees and management with solutions related to workflows, task execution, and performance improvement, thereby strengthening employees’ sense of responsibility for work outcomes (Davenport, 2018) and motivating them to make extra efforts to contribute to team and organizational success. Furthermore, AI’s predictive and analytical capabilities may stimulate employees’ innovative thinking, encouraging them to propose new ideas for improving workflows and organizational operations (Wu & Zhang, 2024). This proactive behavior is a key component of organizational citizenship behavior (Yin et al., 2024). Therefore, this research proposes the following hypotheses:

**Hypothesis 4a.**  *DAU will be positively related to IP.*

**Hypothesis 4b.**  *DAU will be positively related to EP.*

### 2.6. A Serial Mediation Model

Based on the analysis above, as individuals’ AIA increases, POQ serves as an emotional mediator that shapes the influence of AIA on employees’ interactions with AI and their performance. Self-construal theory explains how individuals adjust their behavioral responses based on their self-perceptions and emotions (Hong & Chang, 2015; Markus & Kitayama, 1991; Matsumoto, 1999). Employees with an independent self-construal adopt a more rational and critical attitude, rigorously evaluating the role of AI in supporting their work, thereby fostering RAU. Independent self-construal drives individuals to focus on how AI can better serve their tasks and goals during the reflection process, which enhances IP.

Individuals consciously alter their natural and social environments, but there are differences in their tendencies to initiate such changes (Matsumoto, 1999; Stapel & Koomen, 2001). Employees with POQ are often considered unsuited to their current work environments (Ma et al., 2023; Zhang et al., 2016). However, when faced with the challenges posed by AI technologies, high POQ drives employees to engage in reflective activities on AI usage, demonstrating their ability to break free from environmental constraints and actively reshape their surroundings. Employees with reflective thinking are more motivated to exhibit organizational citizenship behaviors, showing greater care for colleagues and organizations and proactively contributing (Jong & Elfring, 2017; Schippers et al., 2013). RAU indicates employees’ willingness to think critically about AI’s applications in their work, helping them unlock potential, increase engagement, and take ownership by voluntarily performing tasks beyond their formal responsibilities, thereby enhancing EP.

For individuals with a relational self-construal, their focus lies in building and maintaining connections with the external environment, including understanding and forming high-quality relationships with AI (Cross et al., 2011; Stapel & Koomen, 2001). When confronted with the challenges of AI technologies, employees with high POQ leverage their knowledge to adapt to AI, foster continuous interactions, and strengthen their DAU. In the workplace, DAU implies viewing AI as an essential tool to assist in task completion, thereby improving IP.

Furthermore, for individuals with a relational self-construal, AIA triggering POQ may lead to increased DAU. In such cases, employees perceive AI as a valuable resource and an opportunity to adapt to the ever-changing work environment of the digital era. The application of AI in the workplace reduces task costs, enriches employees’ psychological resources, and inspires positive behaviors such as knowledge sharing and helping colleagues, thus enhancing EP. Therefore, this research proposes the following hypotheses:

**Hypothesis 5a.**  *AIA will have a positive indirect effect on IP through the serial mediation of POQ and RAU.*

**Hypothesis 5b.**  *AIA will have a positive indirect effect on EP through the serial mediation of POQ and RAU.*

**Hypothesis 6a.**  *AIA will have a positive indirect effect on IP through the serial mediation of POQ and DAU.*

**Hypothesis 6b.**  *AIA will have a positive indirect effect on EP through the serial mediation of POQ and DAU.*

### 2.7. Employee–AI Collaboration, AI Awareness, and Perceived Overqualification

In recent years, artificial intelligence technology has rapidly advanced and become a significant driving force in promoting technological breakthroughs, industrial structure optimization, and overall productivity growth (Parteka & Kordalska, 2023). On a macro level, its applications have expanded from manufacturing industries to service sectors and knowledge-intensive fields. On a micro level, AI technology has reshaped organizational operations, management models, and employees’ work methods, with EAC becoming increasingly frequent (Budhwar et al., 2023). EAC not only refers to the frequency of collaboration between employees and AI tools but also the degree of cooperation between employees and AI during decision-making processes (Sowa et al., 2021; Wu et al., 2024). Research has shown that the level of collaboration between employees and AI is a key factor influencing employees’ acceptance and adaptability to AI (Pereira et al., 2023). Additionally, this collaboration may significantly affect employees’ perceptions of their own work qualifications (Raisch & Krakowski, 2021).

This study assumes that the degree of employee–AI collaboration is closely related to their attitude towards and perception of AI technology. A higher level of collaboration may lead to a deeper understanding of AI and cause employees to reflect on whether their skills are in line with the development of AI technology. In addition, frequent collaboration could lead employees to become more aware of the risk that their jobs may be replaced by AI, causing them to focus more on whether their skills exceed the requirements of their work. On the other hand, employees who collaborate less with AI may have insufficient understanding and adaptability to AI, leading to greater uncertainty and fear of AI and hindering their ability to assess how well their skills match the job requirements. Therefore, the degree of collaboration between workers and AI could serve as a moderator for the relationship between AIA and POQ. Based on the above literature review, this research proposes the following hypotheses:

**Hypothesis 7.**  *The degree of EAC moderates the relationship between AIA and POQ, such that the positive correlation between AIA and POQ strengthens (weakens) when the level of EAC is higher (lower)*.

## 3. Research Methodology

### 3.1. Research Procedure and Sample

This study collected questionnaire data through the online platform Credamo. Compared to other data collection methods, online platforms offer a more diverse sample pool, flexible questionnaire design capabilities, and easy access to high-quality data. To minimize the impact of common method bias, a three-stage research design was implemented, with data collected at three distinct time points. Specifically, in the first stage, data on AIA, AI collaboration, and participants’ demographic information were collected. A total of 500 questionnaires were distributed, with 400 valid responses returned, resulting in an 80% valid response rate. In the second stage, participants who completed the first-stage survey were invited to participate in the second stage, where data on POQ, RAU, and DAU were collected. A total of 400 questionnaires were distributed, and 372 valid responses were returned, yielding a 93% valid response rate. In the third stage, participants who completed the second-stage survey were invited to take part in the third survey, which primarily collected data on IP and EP. A total of 372 questionnaires were distributed, and after excluding those with clear response patterns or mismatched answers, 353 valid responses were obtained, resulting in a valid response rate of 94.892%. Regarding sample distribution, 48.73% were male (SD = 0.501), with a balanced gender ratio. Participants aged 21–30 numbered 103 (29.18%), those aged 31–40 were 206 (58.36%), and those over 40 numbered 44 (12.46%). Among the participants, 303 (85.84%) held a bachelor’s degree or higher (including master’s and doctoral degrees). Regarding job position, 213 (60.34%) were general employees, 111 (31.44%) were lower-level managers, and 24 (6.80%) were mid-level managers. Regarding company size, 11.90% of participants were from companies with fewer than 50 employees, 22.10% from companies with 51–100 employees, 42.78% from companies with 101–150 employees, 16.71% from companies with 151–200 employees, and 6.52% from companies with more than 200 employees. Table 1 presents the respondents’ demographic features.

### 3.2. Measurement Tools

To ensure the reliability and validity of the survey, measures for AIA, POQ, and performance were adopted from well-established scales published in internationally reputable journals. All scales were meticulously translated into Chinese using a rigorous “translation-back translation” method. A 5-point Likert scale ranging from “strongly disagree” (1) to “strongly agree” (5) was applied to all measurements. The measurement items are presented in Appendix A.

AI Awareness: A 4-item scale developed by Brougham and Haar (Brougham & Haar, 2018) was utilized to assess AI awareness. Example items include statements like, “I believe my job may be replaced by artificial intelligence”. The scale demonstrated high reliability, with a Cronbach’s α of 0.861.

Employee–AI Collaboration: The 5-item scale used to measure employee–AI collaboration was adapted from Kong et al. (Kong et al., 2023), with items such as “AI is involved in my decision-making process”. The scale demonstrated high reliability, with a Cronbach’s α of 0.884.

Perceived Overqualification: The 9-item scale used to measure perceived overqualification was based on Maynard et al. (Maynard et al., 2006), with items such as “The level of education required for my job is lower than my current educational level”. The scale demonstrated high reliability, with a Cronbach’s α of 0.938.

Reflection on AI Usage: The 6-item scale adapted from Somech (2006) was used to measure self-reflection in AI usage, such as “In using AI, I always look for different interpretations and perspectives to approach problems”. The scale demonstrated high reliability, with a Cronbach’s α of 0.903.

Dependence on AI usage: The 3-item scale used to measure dependence on AI usage was from Tang et al. (Tang et al., 2023), with items such as “I rely on AI to handle or assist with work-related activities”. The scale demonstrated high reliability, with a Cronbach’s α of 0.828.

In-role performance: The 5-item task performance scale from Methot et al. (Methot et al., 2016) was used to measure in-role performance, with items such as “I can fully complete the duties assigned to me”. The scale demonstrated high reliability, with a Cronbach’s α of 0.896.

Extra-role performance: The 10-item organizational citizenship behavior scale from Bachrach et al. (Bachrach et al., 2007) was used to measure extra-role performance, with items such as “If an employee’s work is lagging behind, I will offer help”. The scale demonstrated high reliability, with a Cronbach’s α of 0.947.

## 4. Results

### 4.1. Confirmatory Factor Analysis

In this study, AMOS 26.0 was used to conduct confirmatory factor analysis (CFA) on the seven constructs to examine the discriminant validity of the variables. The baseline model, a seven-factor model, was compared with six competing models: a six-factor model (combining AIA and EAC), a five-factor model (combining AIA, EAC, and POQ), a four-factor model (combining AIA, EAC, POQ, and RAU), a three-factor model (combining AIA, EAC, POQ, RAU, and DAU), a two-factor model (combining AIA, EAC, POQ, RAU, DAU, and IP), and a single-factor model (combining all constructs) (Table 2). The comparison revealed that the original seven-factor model exhibited the best fit, indicating that the model used in this study has good discriminant validity.

### 4.2. Common Method Bias

Given that all surveys were completed using self-reported data from employees, potential common method bias (CMB) was a concern. To test for CMB, Harman’s single-factor test was conducted. The results revealed that the eigenvalue of the first factor was greater than 1, and the cumulative percentage of the first factor was 36.956%, which is below the critical threshold of 40% for common method bias. This indicates that common method bias is not a significant issue in this study.

Furthermore, an additional latent factor was added to the seven-factor model (the hypothesized model), creating an eight-factor model. The results showed that the eight-factor model did not significantly improve the fit indices of the seven-factor model, with only marginal changes observed (ΔCFI = 0.007, ΔTLI = 0.007, ΔRMSEA = 0.002). Therefore, common method bias does not pose a threat to the results of this study. This suggests that AIA, EAC, POQ, RAU, DAU, IP, and EP represent seven distinct constructs, allowing for further analysis in subsequent research.

### 4.3. Descriptive Statistics and Correlation Analysis

Table 3 summarizes the means, standard deviations, and correlation coefficients for the variables included in the model. As shown in Table 3, AIA is significantly positively correlated with POQ (r = 0.395, *p* < 0.01). POQ is also significantly positively correlated with DAU (r = 0.444, *p* < 0.01) and RAU (r = 0.450, *p* < 0.01). DAU shows a positive correlation with IP (r = 0.460, *p* < 0.01) and EP (r = 0.495, *p* < 0.01). Similarly, RAU is positively correlated with IP (r = 0.445, *p* < 0.01) and EP (r = 0.430, *p* < 0.01). These results provide preliminary support for the main variable relationships in the chain mediation model of this study.

### 4.4. Hypothesis Testing

#### 4.4.1. Direct Effects Testing

Table 4 displays the results of the direct effects analysis conducted on the full sample. After controlling for demographic characteristics such as gender, age, education level, job position, and company size, it was found that AIA is significantly positively correlated with POQ (β = 0.388, *p* < 0.01), thus confirming Hypothesis 1. This suggests that when employees have higher AIA, they perceive that the application of AI technology has impacted their work, leading to a significant increase in POQ. Additionally, POQ is significantly positively correlated with RAU (β = 0.450, *p* < 0.01) and DAU (β = 0.488, *p* < 0.01). Hypotheses 2a and 2b are supported. RAU is significantly positively correlated with IP (β = 0.487, *p* < 0.01) and EP (β = 0.460, *p* < 0.01). Hypotheses 3a and 3b are supported. DAU is significantly positively correlated with IP (β = 0.450, *p* < 0.01) and EP (β = 0.466, *p* < 0.01). Hypotheses 4a and 4b are supported. These results provide preliminary support for the main variable relationships in the mediation model of this study.

#### 4.4.2. Mediation Effects Testing

As shown in Table 5, the mediation effect of POQ between AIA and DAU is significant (β = 0.139, 95% CI = [0.081, 0.183]). Similarly, the mediation effect of POQ between AIA and RAU is also significant (β = 0.137, 95% CI = [0.093, 0.192]). Furthermore, the multiple mediation effect of AIA through POQ and RAU on IP is significant (β = 0.036, 95% CI = [0.017, 0.057]), as is the effect on EP (β = 0.032, 95% CI = [0.015, 0.052]). Hypotheses 5a and 5b are supported. The multiple mediation effect of AIA through POQ and DAU on IP is significant (β = 0.035, 95% CI = [0.017, 0.056]), as is the multiple mediation effect on EP (β = 0.040, 95% CI = [0.021, 0.062]). Hypotheses 6a and 6b are supported.

#### 4.4.3. Moderating Effect Testing

**Figure 2:**
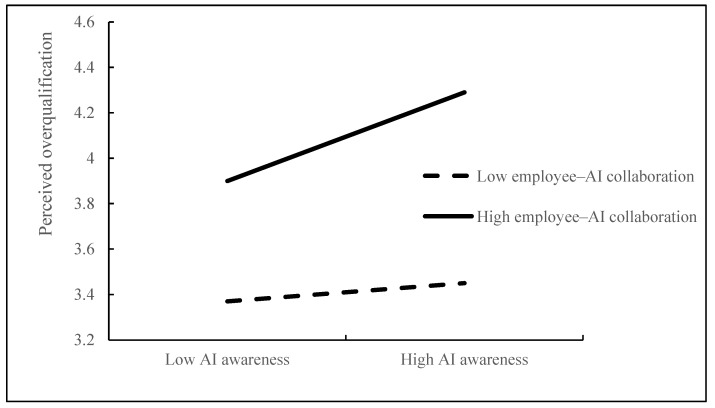
The moderating effect of employee–AI collaboration.

Table 6 illustrates that the interaction between AIA and EAC exerts a significant positive influence on POQ (β = 0.166, *p* = 0.01). Hypothesis 7 is supported. This suggests that the impact of AIA on POQ varies significantly depending on the level of EAC. To more clearly illustrate the moderating effect of EAC between AIA and POQ, we conducted a simple slope analysis, as shown in Figure 2. When EAC is at a higher level, the positive correlation between AIA and POQ becomes more pronounced; conversely, when EAC is at a lower level, this relationship weakens.

## 5. General Discussion

This study, based on a sample of 353 employees and drawing from self-construal theory, explores the chain mediation model proposed in this paper, revealing the two distinct pathway mechanisms and boundary conditions through which employees’ AIA, mediated by POQ, influences IP and EP under the impact of AI technology. The key findings are as follows: (1) Employees’ AIA leads to POQ, which, in turn, triggers reflection on DAU. (2) Employees’ AIA positively influences their dependence on and RAU through POQ, thus affecting both IP and EP. (3) When EAC is at a higher level, employees’ AIA is more likely to trigger POQ, leading to cognitive and emotional escalations that subsequently result in different responses to AI. The degree of interaction between employees and AI serves as a key event, prompting employees to assess the work transformation brought about by AI technology and their own circumstances. The following section summarizes the specific contributions of this study and its practical implications.

### 5.1. Theoretical Contributions

First, this study enriches the research framework on AI applications. Currently, the application of artificial intelligence in organizations is still in its early stages, and there is limited literature on AIA. Moreover, existing studies on the consequences of AIA present contradictory findings. Early research suggested that AIA negatively impacts employees by inducing job insecurity, reducing organizational commitment, depleting psychological resources, and leading to burnout and emotional exhaustion (Brougham & Haar, 2018; Kong et al., 2021; Liang et al., 2022). More recent scholars, however, have argued that AIA could trigger employees to engage in job crafting behaviors, implementing more work redesigns to enhance personal competitiveness (Ding, 2021). This study conducts an empirical analysis of the relationships among AIA, POQ, RAU, DAU, IP, EP, and EAC. The findings reveal that AIA significantly influences both IP and EP, reinforcing the utility value of AIA proposed in previous research and affirming the positive impact of AI on human development (Huang & Rust, 2018).

Secondly, POQ, as one of the most important psychological states of employees (Erdogan & Bauer, 2021), provides a new perspective for the causal relationships explored in this study. It establishes a significant connection between the literature on AIA and AI usage behaviors in the workplace. Extensive research demonstrates that employees’ perceptions of person–job fit influence their behaviors and outcomes (P. Liu et al., 2025; Ma et al., 2023). When employees perceive that the introduction of AI into their work environment renders their knowledge and skills “overqualified” relative to job requirements (Kong et al., 2021), POQ emerges. Studies suggest that POQ can yield positive outcomes, such as creativity (Luksyte & Spitzmueller, 2016), speaking up (S. Liu et al., 2024), and organizational citizenship behaviors (Pandey et al., 2023). Within the framework of self-construal theory, this study innovatively uses POQ as an explanatory mechanism for the different behaviors triggered by AIA in employees, thereby expanding its application boundary. Furthermore, it enriches the conclusions drawn from multiple theoretical perspectives in this field. The results also suggest that examining the alignment between employees’ qualifications and job requirements may be an effective way to trigger AI usage reflection and dependence.

Thirdly, this study finds that EAC is a crucial enabling condition for AIA and also critically examines the limitations of AIA. Existing research has emphasized the interaction between the individual, environment, and occupation and their impact on job performance (Bass et al., 2024; Kristof-Brown et al., 2005). This study examines the phenomenon within the specific context of human–AI interaction, where the use of new technologies is found to trigger employees’ latent perceptions, such as robot trust (Wu & Zhang, 2024), technology anxiety (Chen et al., 2024), and perceived substitution crises (Wu et al., 2024). The study demonstrates that EAC strengthens the relationship between AIA and POQ, providing a valuable supplement to the mainstream perspective and contributing new insights to research on the hidden outcomes of new technologies.

### 5.2. Practical Implications

This research also provides several important managerial implications. First, the chain mediation model in this research suggests that organizations should pay more attention to employees’ AIA, POQ, and other cognitive and emotional factors in the workplace. These intangible factors are often overlooked but are a significant source of performance differences among employees. Therefore, we recommend that organizations design appropriate employee feedback channels based on their own size and resources. For small organizations, one-on-one interviews, regular team meetings, and online surveys can be implemented to collect employee questions and suggestions on the use of AI, while for large organizations, a combination of internal platforms, project management tools, and anonymous survey tools can be used to collect employee feedback on AI applications and suggestions. These measures would not only enhance communication and collaboration among employees but also facilitate the timely understanding of changes in employees’ AIA and guide organizations in designing and applying AI technologies that better align with job requirements, thereby maximizing AI’s potential in cost reduction and efficiency enhancement.

Secondly, as AI applications become increasingly widespread in organizations (Saydam et al., 2022), we need to consider how to build new relationships that allow humans and machines to work collaboratively and complement each other’s strengths. Employees’ interactions with AI in the workplace affect how they perceive and evaluate the match between their qualifications and their job. In today’s environment, where competition is instantaneous, employees’ perceptions of external technological changes are crucial. Enterprises should focus on “how to enable employees to better utilize AI and let AI serve human development” and strive to create a “challenge-skill balance” work environment. Organizations should place emphasis on the alignment of employees’ qualifications with job requirements and focus on training and developing their potential knowledge and skills. This would improve the overall dynamic adaptability of the workforce, ensure the sustainable growth of the organization, and avoid threatening employees’ role efficiency, reducing role ambiguity, or even replacing their work.

### 5.3. Limitations and Future Research

This study has several limitations. Due to constraints in time and geography, there are certain limitations to the survey sample. The data for this study is sourced from China, where the prevalence of artificial intelligence in workplaces remains lower than in developed countries, potentially limiting the generalizability of the findings. Future research should incorporate data from diverse countries and economic contexts to validate the findings.

Second, the data in this study is based on self-reports from individuals, which may be subject to social desirability bias (Podsakoff et al., 2003). To address this limitation, future research could incorporate multi-source data collection methods, such as having supervisors or managers provide assessments of EAC, IP, and EP. This approach could enhance the objectivity and robustness of the findings, offering a more comprehensive understanding of these relationships.

Third, in examining boundary conditions, this study only explored a single factor, namely AI collaboration. However, existing research on AI has identified various forms of human–AI interaction, such as embedded AI, virtual AI, and robotic AI (Glikson & Woolley, 2020), all of which could also impact the results. Future research could consider incorporating a wider range of factors to explore the boundary mechanisms of AIA.

Finally, research suggests that the application of AI in the workplace may serve as a source of stress for employees (Wu et al., 2024). Future studies could further explore individual differences in employees’ cognitive appraisals of stress, such as challenge appraisal and threat appraisal, which may influence variations in their work behaviors.

## 6. Conclusions

In the context of the continuous development of STARA (Smart Technologies, Automation, Robotics, and Artificial Intelligence) and the intensifying issue of employment “involution”, human–machine symbiosis is gradually becoming a new organizational form and a new work scenario for employees (Brougham & Haar, 2018). Furthermore, with the global economic downturn, the contradiction between AI-driven cost reduction and efficiency improvement and employees’ sustainable career development has become increasingly pronounced. To address this issue, companies must guide employees toward a healthy understanding of AI, unlocking their potential to reduce labor costs, increase skill diversity, and alleviate competition in saturated markets. This study provides an in-depth analysis of the mechanisms through which employees’ AIA functions, revealing how it enables the release of employees’ knowledge and skills, allowing them to grow and adapt to the process of AI interaction. Employees’ AIA tends to generate a higher sense of POQ, which subsequently leads to increased DAU and RAU, resulting in performance growth in both in-role and extra-role dimensions. EAC is a crucial precursor to triggering POQ and its subsequent effects.

## Figures and Tables

**Table 1:** Participant profile.

Variables	Category	N = 353	%
Gender	Male	172	48.73
	Female	181	51.27
Age	21–30 years	103	29.18
	31–40 years	206	58.36
	Above 40 years	44	12.46
Education	High school	7	1.98
	Associate degree	53	15.01
	Bachelor’s degree	80	22.66
	Master’s degree	147	41.64
	Doctorate	76	21.53
Position	Regular employee	213	60.34
	Junior manager	111	31.44
	Middle manager	24	6.80
	Senior manager	5	1.42
Size	50 employees or fewer	42	11.90
	51–100 employees	78	22.10
	101–150 employees	151	42.78
	151–200 employees	59	16.71
	Above 200 employees	23	6.52

**Table 2:** Confirmatory Factor Analysis Test Results of Variables (N = 353).

Model	X^2^/df	RMSEA	CFI	GFI	IFI	TLI
Seven-factor model (AIA, EAC, POQ, RAU, DAU, IP, EP)	1.088	0.016	0.993	0.898	0.993	0.992
Six-factor model (AIA + EAC, POQ, RAU, DAU, IP, EP)	1.651	0.043	0.945	0.825	0.946	0.942
Five-factor model (AIA + EAC + POQ, RAU, DAU, IP, EP)	2.619	0.068	0.863	0.705	0.864	0.855
Four-factor model (AIA + EAC + POQ + RAU, DAU, IP, EP)	3.584	0.086	0.781	0.601	0.782	0.768
Three-factor model (AIA + EAC + POQ + RAU + DAU, IP, EP)	3.878	0.090	0.755	0.579	0.756	0.742
Two-factor model (AIA + EAC + POQ + RAU + DAU + IP, EP)	4.556	0.101	0.697	0.536	0.698	0.681
One-factor model (AIA + EAC + POQ + RAU + DAU + IP + EP)	6.133	0.121	0.562	0.429	0.563	0.539

**Table 3:** Means, Standard Deviations, and Correlation Coefficients of Variables (N = 353).

Variables	M	SD	1	2	3	4	5	6	7	8	9	10	11	12
1. Gender	1.487	0.501	1											
2. Age	1.833	0.624	−0.002	1										
3. Education	2.904	1.214	−0.053	−0.010	1									
4. Position	1.592	0.492	−0.021	−0.010	−0.056	1								
5. Size	2.538	1.636	−0.030	0.063	0.006	−0.048	1							
6. AIA	3.309	0.973	0.044	−0.002	−0.068	−0.014	0.053	1						
7. EAC	3.336	0.959	−0.026	0.019	−0.042	−0.006	−0.006	0.430 **	1					
8. POQ	3.412	0.951	−0.057	−0.057	−0.014	0.036	0.015	0.395 **	0.433 **	1				
9. RAU	3.314	1.049	−0.047	0.026	−0.045	0.002	0.029	0.433 **	0.412 **	0.444 **	1			
10. DAU	3.356	0.955	−0.088	−0.030	−0.097	0.031	−0.116 *	0.383 **	0.431 **	0.450 **	0.485 **	1		
11. IP	3.345	1.011	−0.042	0.014	0.022	0.016	0.023	0.434 **	0.492 **	0.438 **	0.460 **	0.445 **	1	
12. EP	3.344	0.988	0.023	0.024	−0.031	0.038	−0.025	0.455 **	0.498 **	0.425 **	0.495 **	0.430 **	0.475 **	1

Note: ** *p* < 0.01, * *p* < 0.05.

**Table 4:** Direct effect regression analysis (N = 353).

Variables	POQ	RAU	DAU	IP		EP	
Constant	2.313 ** (5.024)	2.062 ** (4.747)	1.945 ** (4.009)	1.031 * (2.207)	1.157 * (2.533)	1.805 ** (3.919)	1.772 ** (4.035)
Control Variable							
Gender	−0.146 (−1.556)	−0.125 (−1.381)	−0.054 (−0.535)	0.007 (0.067)	−0.030 (−0.310)	0.115 (1.198)	0.085 (0.917)
Age	−0.104 (−1.283)	0.024 (0.328)	0.071 (0.856)	0.057 (0.711)	0.037 (0.463)	0.031 (0.395)	0.011 (0.144)
Education	0.024 (0.504)	−0.072 (−1.935)	−0.038 (−0.916)	0.055 (1.355)	0.037 (0.918)	0.011 (0.270)	−0.004 (−0.116)
Position	0.007 (0.098)	0.044 (0.616)	−0.024 (−0.296)	0.117 (1.518)	0.149 (1.950)	−0.050 (−0.659)	−0.019 (−0.256)
Size	−0.029 (−0.635)	−0.072 * (−2.570)	0.015 (0.490)	0.043 (1.419)	0.001 (0.033)	0.018 (0.610)	−0.022 (−0.778)
Independent variable							
AIA	0.388 ** (7.924)						
Mediating variable							
POQ		0.450 ** (9.442)	0.488 ** (9.177)				
RAU				0.487 ** (9.434)		0.460 ** (9.052)	
DAU					0.450 ** (9.750)		0.466 ** (10.515)
R^2^	0.170	0.266	0.208	0.215	0.226	0.201	0.252
Adjusted R^2^	0.149	0.245	0.187	0.194	0.205	0.180	0.232
F	7.825 **	12.393 **	10.025 **	10.424 **	11.104 **	9.597 **	12.812 **

Note: ** *p* < 0.01, * *p* < 0.05; values in parentheses are standard errors.

**Table 5:** Mediation Effect Test Results (N = 353).

Path	Effect	Boot SE	BootLLCI	BootULCI
AIA → POQ → DAU	0.139	0.026	0.081	0.183
AIA → POQ → RAU	0.137	0.025	0.093	0.192
AIA → POQ → DAU → IP	0.035	0.010	0.017	0.056
AIA → POQ → DAU → EP	0.040	0.010	0.021	0.062
AIA → POQ → RAU → IP	0.036	0.010	0.017	0.057
AIA → POQ → RAU → EP	0.032	0.010	0.015	0.052

**Table 6:** Moderation effect analysis (N = 353).

Variables	POQ		
Constant	3.596 ** (8.635)	3.693 ** (9.326)	3.669 ** (9.413)
Control Variable			
Gender	−0.146 (−1.556)	−0.116 (−1.299)	−0.118 (−1.338)
Age	−0.104 (−1.283)	−0.109 (−1.415)	−0.118 (−1.566)
Education	0.024 (0.504)	0.013 (0.289)	0.016 (0.365)
Position	0.007 (0.098)	−0.006 (−0.081)	−0.012 (−0.179)
Size	−0.029 (−0.635)	−0.035 (−0.809)	−0.029 (−0.697)
Independent variable			
AIA	0.388 ** (7.924)	0.252 ** (4.890)	0.231 ** (4.539)
Moderating variable			
EAC		0.318 ** (6.168)	0.275 ** (5.275)
Interaction effect			
AIA × EAC			0.166 ** (3.458)
R^2^	0.17	0.253	0.279
Adjusted R^2^	0.149	0.232	0.255
F	7.825 **	11.607 **	11.976 **

Note: ** *p* < 0.01; values in parentheses are standard errors.

## Data Availability

The original contributions presented in this study are included in the article. Further inquiries can be directed to the corresponding author.

## Appendix A

The following are the survey items.

AI awareness:

1. I think my job could be replaced by STARA.
2. I am personally worried that what I do now in my job will be able to be replaced by STARA.
3. I am personally worried about my future in my organization due to STARA replacing employees.
4. I am personally worried about my future in my industry due to STARA replacing employees.

Employee–AI collaboration:

1. AI participates in my decision-making process.
2. AI participates in my prediction process.
3. AI participates in my problem-solving process.
4. AI participates in my information identification and evaluation process.
5. AI participates in my problems, opportunities, or risk recognition process.

Perceived Overqualification:

1. My job requires less education than I have.
2. The work experience that I have is not necessary to be successful on this job.
3. I have job skills that are not required for this job.
4. Someone with less education than myself could perform well on my job.
5. My previous training is not being fully utilized in this job.
6. I have a lot of knowledge that I do not need in order to do my job.
7. My education level is above the education level required for my job.
8. Someone with less work experience than myself could do my job just as well.
9. I have more abilities than I need in order to do my job.

Reflection on AI Usage:

1. In my use of AI, I always look for different interpretations and perspectives to confront problems.
2. In my use of AI, I critically examine AI’s work in order to improve my work efficiency.
3. In my use of AI, I am prepared to reflect on the way I utilize AI.
4. In my use of AI, I engage in evaluating my weaknesses in utilizing AI to achieve efficiency.
5. In my use of AI, I openly challenge the perspectives proposed by AI.
6. In my use of AI, I reassess any proposed AI application solutions.

Dependence on AI Usage:

1. I depended on AI to handle or help with work-related activities.
2. I depended on AI to make major work-related decisions.
3. I depended on AI to check and monitor my work.

In-role performance:

1. I can adequately complete assigned duties.
2. I can fulfill the responsibilities specified in my job description.
3. I can perform tasks that are expected of me.
4. I can meet the formal performance requirements of the job.
5. I can engage in activities that will directly affect my performance evaluations.

Extra role performance:

1. Help other employees out if someone falls behind in his/her work.
2. Willingly share expertise with other members of the unit.
3. Try to act like a peacemaker when other unit members have disagreements.
4. Take steps to try to prevent problems with other unit members.
5. Willingly give time to help unit members who have work-related problems.
6. ‘Touch base’ with other unit members before initiating actions that might affect them.
7. Encourage other unit members when someone is down.
8. Provide constructive suggestions about how the unit can improve its effectiveness.
9. Be willing to risk disapproval to express beliefs about what’s best for the unit.
10. Attend and actively participate in team meetings.
